# Implementation of artificial intelligence algorithms for melanoma screening in a primary care setting

**DOI:** 10.1371/journal.pone.0257006

**Published:** 2021-09-22

**Authors:** Mara Giavina-Bianchi, Raquel Machado de Sousa, Vitor Zago de Almeida Paciello, William Gois Vitor, Aline Lissa Okita, Renata Prôa, Gian Lucca dos Santos Severino, Anderson Alves Schinaid, Rafael Espírito Santo, Birajara Soares Machado

**Affiliations:** Image Research Center, Hospital Israelita Albert Einstein, São Paulo, SP, Brazil; Taipei Medical University, TAIWAN

## Abstract

Skin cancer is currently the most common type of cancer among Caucasians. The increase in life expectancy, along with new diagnostic tools and treatments for skin cancer, has resulted in unprecedented changes in patient care and has generated a great burden on healthcare systems. Early detection of skin tumors is expected to reduce this burden. Artificial intelligence (AI) algorithms that support skin cancer diagnoses have been shown to perform at least as well as dermatologists’ diagnoses. Recognizing the need for clinically and economically efficient means of diagnosing skin cancers at early stages in the primary care attention, we developed an efficient computer-aided diagnosis (CAD) system to be used by primary care physicians (PCP). Additionally, we developed a smartphone application with a protocol for data acquisition (i.e., photographs, demographic data and short clinical histories) and AI algorithms for clinical and dermoscopic image classification. For each lesion analyzed, a report is generated, showing the image of the suspected lesion and its respective Heat Map; the predicted probability of the suspected lesion being melanoma or malignant; the probable diagnosis based on that probability; and a suggestion on how the lesion should be managed. The accuracy of the dermoscopy model for melanoma was 89.3%, and for the clinical model, 84.7% with 0.91 and 0.89 sensitivity and 0.89 and 0.83 specificity, respectively. Both models achieved an area under the curve (AUC) above 0.9. Our CAD system can screen skin cancers to guide lesion management by PCPs, especially in the contexts where the access to the dermatologist can be difficult or time consuming. Its use can enable risk stratification of lesions and/or patients and dramatically improve timely access to specialist care for those requiring urgent attention.

## 1. Introduction

Skin cancer is currently the most common type of cancer among Caucasians [1]. Approximately 1 in 5 Americans will develop skin cancer at some point in their lives [2]. A continuous increase in skin cancer rates is being observed [1, 3]. Keratinocytic cancers (KCs) are the most common type, with a relative incidence increase of up to 10% per year, with 2–3 million new cases each year worldwide [4]. Among KCs, basal cell carcinoma (BCC) and cutaneous squamous cell carcinoma (cSCC) are the most frequent types [5]. Melanoma, although less frequent, is responsible for more than 95% of the deaths by skin cancer [6].

The increase in life expectancy and population ageing along with the introduction of in vivo diagnostic tools (e.g. dermoscopy) and new treatments for advanced melanoma and KCs have resulted in unprecedented changes in the organization of patient care in the clinical setting [7]. This generates a great burden on national healthcare systems and economies. In a recent study from Australia, the mean annual cost per patient for melanoma stage 0/I/II was US$1,175, rising to US$80,440 for stage III/IV [8]. In addition, the treatment of basal cell carcinoma and squamous cell carcinoma in 2011 in the United States was associated with costs of up to US$4.8 billion [9]. In our country, there are very few studies about health cost in general, especially those regarding to skin cancers.

Secondary prevention encompasses the various strategies of early detection of skin tumors, including skin cancer screening programs. These strategies are expected to reduce the epidemiologic burden and the cost of skin cancer management–under the premise that skin cancer can be more easily and effectively treated at an early phase of development [7]. Dermatologists are the best trained and equipped specialists to provide early detection, but their success depends on patients receiving timely access to their services. In some countries, the lack of dermatologists has led to an increase in use of e-Health and teledermatology over the last 10 years [7]. Sensitivity has been found to be comparable between teledermatology associated with the use of dermoscopy and face-to-face evaluations, although specificity was higher in face-to-face evaluations [10]. Another instrument being studied for early detection is the use of artificial intelligence (AI) algorithms as a support decision system for suspected skin cancer lesions. Many studies have demonstrated that AI can perform at least as well as dermatologists evaluating dermoscopy images of skin cancer [11–13].

Brazil is a large country [14] with 212 million inhabitants as of 2020 [15]. Since 1988, Brazil’s constitution assures that the government has to provide free health care to its population and to foreigners while in the country [16]. More than 70% of the population depend exclusively on the public healthcare system [17], which includes 42,488 public primary care units [18]. However, dermatologists are poorly distributed around the country [19]. Only 9.1% of the cities in Brazil have practicing dermatologists. This leads patients that live far from large urban centers to travel long distances to have access to dermatologists. Due to the high demand for these professionals, even in big cities, the waiting time for an appointment can be more than 6 months [20].

Recognizing the need for clinically and economically efficient modes of diagnosing skin cancers at early stages in a primary care setting, we conducted this research to develop an efficient computer-aided diagnosis (CAD) system to be used by primary care physicians (PCP) in the primary care centers in Brazil. The goal, set by our founder, was to detect melanoma from other lesions, and not to give the clinician a range of differential diagnosis. In years to come, we plan to expand the range of diagnosis, especially to KCs.

## 2. Methods

This study was approved by the Ethics Committee of Hospital Israelita Albert Einstein (HIAE; CAAE:32903120.4.0000.0071) and it was conducted in the Research Center for Medical Images, at the same hospital, from July 2019-December 2020. Informed consent patient was waived due to the fact that clinical dataset obtained from from our Teledermatology project was anonymized and retrospective. Other datasets used in the study are publicly available.

**Fig 1:**
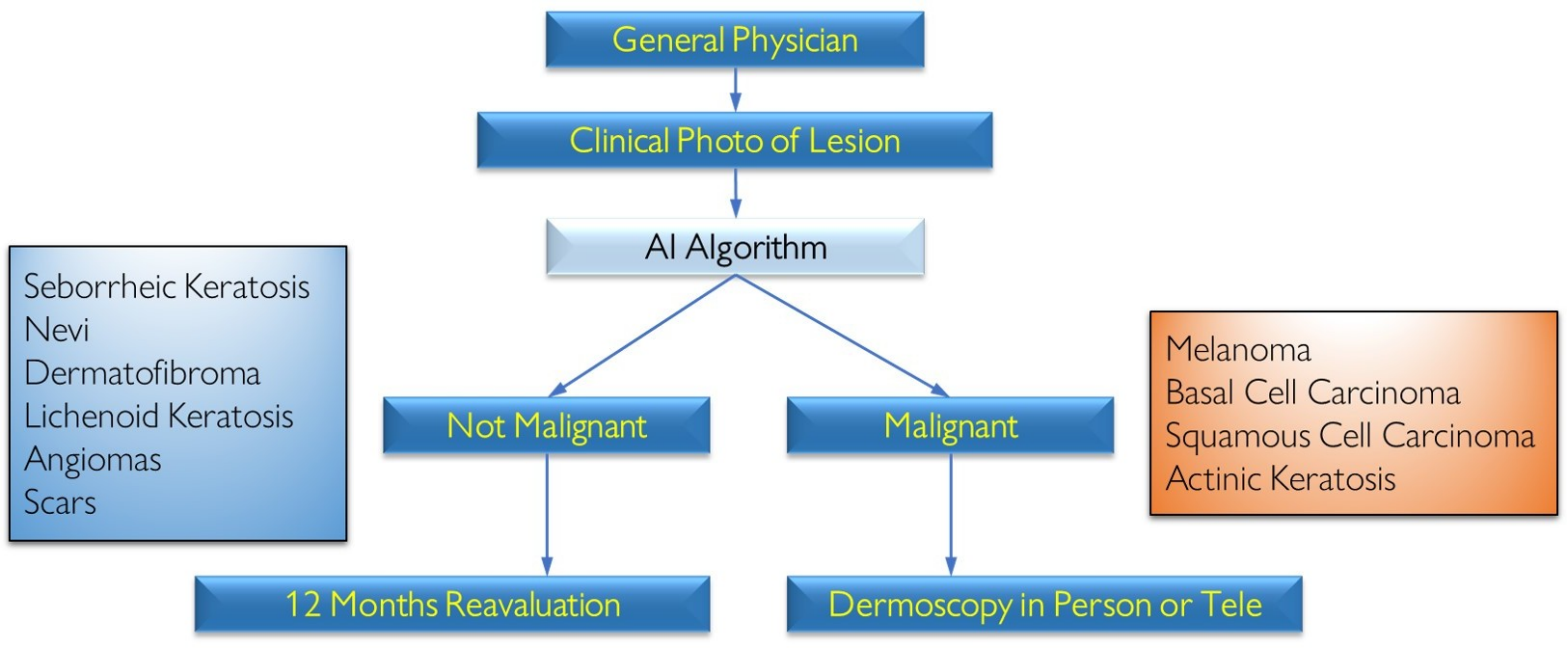
Patient flow for clinical images taken by primary care physicians and possible outcomes and suggested management. If the lesion was suspected to be benign, the management suggested it is to reassess the lesion in 12 months. If the lesion was suspected to be malignant, the management would depend if the PCP had access to the dermoscopy image. If yes, the image would be sent to a dermatologist (Teledermoscopy). If not, the PCP would have to send the patient for in-person dermoscopy.

To develop the CAD system for skin cancer screening, we assessed the main challenges and necessities faced by physicians in five primary care centers. These needs were mostly related to internet connection, app usability, adherence to photography protocols and dermoscopy use. We also did an informal survey with 10 PCPs to have an idea of the profile of this professional. The questionnaire and its main results are described in S1 File. In the next sections, we explore the design of our patient flow in primary care centers (Fig 1), approaching the solutions we found to deal with each of these challenges.

### 2.1 Patient Data Acquisition

We developed an Android and iOS-compatible application to be used by PCPs. The inclusion criteria are: patients 18 years old and the presence of one or more suspicious malignant lesions or its differential diagnosis, such as: melanoma, atypical melanocytic nevus, typical melanocytic nevus, seborrheic keratosis, dermatofibroma, basal cell carcinoma, squamous cell carcinoma, actinic keratosis, viral warts, lichenoid keratosis, hypertrophic scars and pyogenic granuloma. No exclusions criteria will be applied. The protocol for data acquisition in the app will consist of taking clear photographs of as the following: 1) around 50 cm away from the lesion, 2) 10–15 cm away from the lesion and 3) an extra picture with a dermoscopy, if available. Additional patient data that could help a remote specialist to diagnose the lesion includes: demographic data (sex, age, self-declared race), Fitzpatrick’s skin phototype, comorbidities, medications in use, sun habits, previous personal or family history of skin cancer, frequency of sunscreen use, bleeding or pruritus on the lesion, time since the onset of the lesion, and a short clinical history. The app is also capable of storing all this data until an internet connection is available.

## 3. Experiments

### 3.1 AI modelling

**Fig 2:**
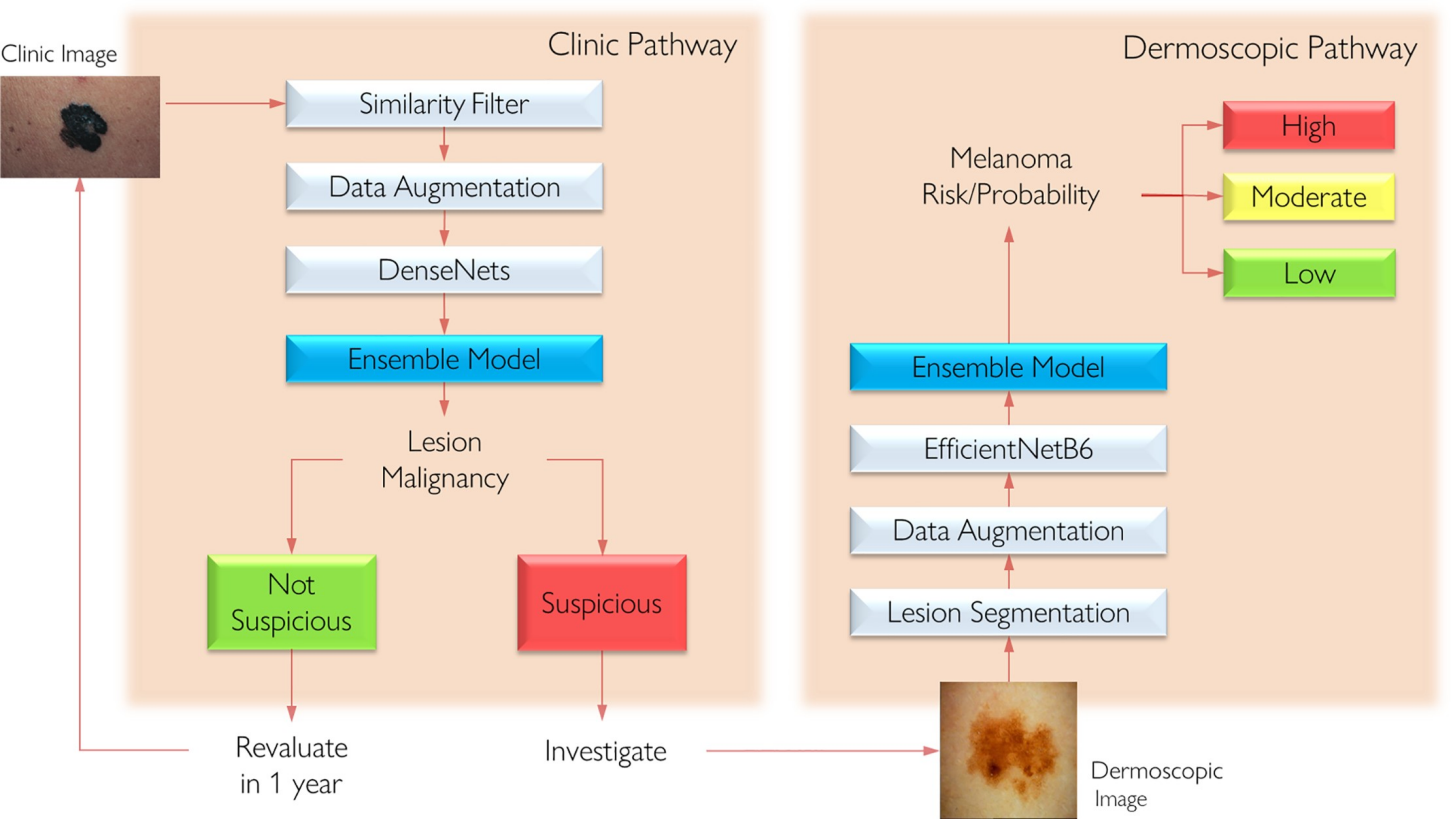
CAD system pathways developed to provide outputs for clinical and dermoscopy images to primary care physicians.

We designed our CAD system to orient the PCP and improve patient management. Since the majority of the public primary care centers do not have access to dermoscopy devices, we trained two models for melanoma screening: one for dermoscopy images (taken with the aid of dermoscopy devices) and another for clinical images (taken directly with the smartphones). The clinical model, which deals with lower-quality images, detects if a lesion is suspicious of malignancy or non-suspicious. The dermoscopy model is supposed to detect if a lesion has as high, moderate or low probability of being a melanoma (Fig 2). In the next sections, we detail how both models were trained.

#### 3.1.1 Dermoscopy model

To train this model we used 26,342 images from the ISIC2019 [13, 21, 22] and PH2 [23] datasets (Table 1). Since these datasets were unbalanced, we created 4,000 additional artificial melanoma images using Generative Artificial Networks (GANs). For this task, we chose StyleGAN2 [24] because it allows us to generate images with a high resolution. Furthermore, to exclude any type of noise that could impair model training and image classification, all the images were cropped by a segmentation model. This model consisted of a MaskRCNN algorithm [25]. Two dermatologists (MGB and ALO) annotated 2000 images using the COCO annotator tool [26]. We randomly split these images into 3 groups, training (80%), validation (10%) and testing (10%). Model training occurred for 20 epochs with 1000 steps per epoch, 20 steps validation, learning rate equals 0.001 and a ResNet101 backbone.

**Table 1:** General view of clinical and dermoscopic algorithms development.

Steps in the algorithm development	Description of the data utilized	Clinical images utilized (n)	Dermoscopic images utilized (n)	Complementary information
Datasets accessed to prepare the final Dataset	Derm-7pt	989	1011	http://derm.cs.sfu.ca/Welcome.html *
	PH2	0	200	https://www.fc.up.pt/addi/ph2%20database.html *
	SD-198	6584	0	https://drive.google.com/file/d/1YgnKz3hnzD3umEYHAgd29n2AwedV1Jmg/view *
	ISIC2019	0	25331	https://www.isic-archive.com/#!/topWithHeader/wideContentTop/main *
	Dermatology Atlas	10972	0	http://www.atlasdermatologico.com.br/index.jsf *
	Sao Paulo City/ HIAE Teledermatology Project	140446	0	Private data (only 141 cases confirmed by histopathological report were actually used to build the CAD system)
Final Dataset utilized for CNN	Total Images (n)	3083	26342	220 x 220 pixels each for dermatoscopic images
				224 x 224 pixels for clinical images
	Training images (n)	2466 (80%)	21074 (80%)	
	Validation Images (n)	309 (10%)	2635 (10%)	
	Test Images (n)	308(10%)	2633 (10%)	
Data augmentation (DAU)	Data generation conventional	No	Yes	19010 melanoma images
	In-place	Yes	Yes	
	GANs	No	Yes	4000 melanoma images
Algorithm Development	Melanoma/non-melanoma (%)	18/82	18/82	
	Similarity filter	Yes	No	TripetLoss/ VGG16
	Segmentation	No	Yes	MaskRCNN
	Modeling	DenseNet	EfficientNetB6	
	Ensemble model	Yes	Yes	Detailed in S1 Table
	Threshold	0.5	0.5	
	Output	Suspicious of malignant lesion:	Suspicious of melanoma:	
			Yes or No	
		Yes or No		
	Visual Explanations	Grad-CAM	Grad-CAM	

* Clicking on the datasets links, all the different diseases that were used in the development of the algorithms will be presented.

We included malignant tumors, such as melanoma, BCC and SCC; premalignant lesions, such as AK; and metastatic carcinomas. For benign tumors, we included melanocytic nevus (all types), blue nevus, vascular lesions, dermatofibroma, epidermal nevus, benign keratosis, soft fibromas, fibromas, keloid, keratoacanthoma, lipoma, epidermoid cyst.

For the classification step, we used an ensemble model that combines the results of five EfficientNetB6 models [27] to get the final output probability. All images on the dataset were resized to 220 x 220 pixels and were randomly partitioned on training (80%), validation (10%) and testing (10%). We used the PH2 dataset only for generalization testing. We trained each EfficientNetB6 model with a different data augmentation technique (S1 Table). For each type of data augmentation, we carried out a training of several networks while varying the optimizers used (rmsprop, adam, sgd). We then selected the model with the best accuracy and sensitivity. To calculate the weights for each class in the dataset, we used the focal loss [28], for unbalanced datasets. A sigmoid function output was chosen to predict the target classes. The ensemble was completed by calculating the mean and the maximum value of the models. A threshold of 0.5 was used for all models [29]. A lesion presenting a mean score above the threshold for all models was considered high probability of melanoma, while a lesion with mean and maximum scores below the threshold was considered a low probability of melanoma. As for the lesions with a maximum score below 0.5 and a mean score above it, they were classified as moderate probability of melanoma diagnosis.

#### 3.1.2 Clinical model

Since 2017, our group has had access to more than 100,000 clinical images of the HIAE’s teledermatology project [20]. Despite the protocols available and an app guiding the PCPs to collect the data, several pictures in the dataset contain artifacts, such as clothes, rings or even showing parts of the office’s furniture. To deal with this variability in the dataset, which could impair AI training and lesion recognition, we trained an algorithm based on similarity networks to filter the images that do not have what we considered the minimum quality for the AI classification. This algorithm classifies the images into seven classes, one that we considered “ideal” images and six others representing each type of the most common artifacts or body parts in the dataset. When the algorithm identifies the image as “ideal”, the classification occurs, but if the image is identified as belonging to any other group, the process is stopped and a message can be sent to the PCP requesting another picture.

This similarity filter consists of a model that extracts features from the images with a convolutional network and classifies these features into the group that has the most similar images to them. Here, we used the VGG16 network [30] with the addition of a dense layer to extract 1024 features from each image and a KNearestNeighbor algorithm with 5 neighbors to classify the image. We selected 14,000 of the clinical images belonging to seven classes (2,000 per class), which we split into 90% for training and 10% for testing. The VGG16 weights were trained with the TripletSemiHardLoss function [31], 0.001 learning rate and a batch size of 32.

**Fig 3:**
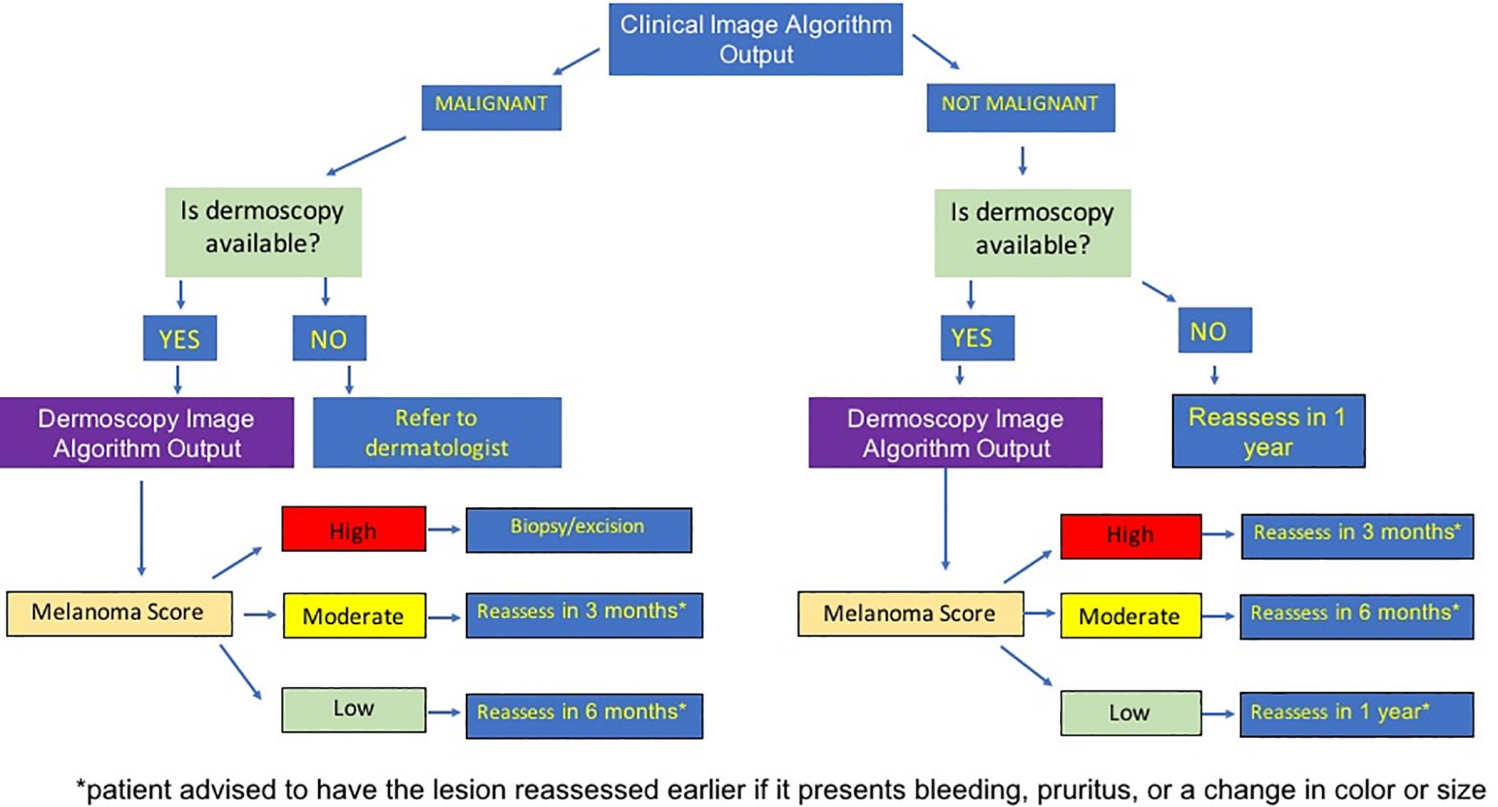
Possible clinical and dermoscopy algorithms outputs and suggested care for each scenario: The second tab was entitled: “Understanding the Heat Map”, with a brief explanation on how to interpret the Heat Map. The third tab has a short text about how uncertainty is inherent to AI algorithms and the fourth and last tab “Who we are” describes the multidisciplinary work group that developed the algorithm. More information about these tabs can be found in S2 File.

The classification ensemble model in the clinical pipeline classifies the “ideal” images in a binary output, suspicious of being malignant or not, considering a threshold of 0.5 and the corresponding suggested management is available in Fig 3. Since biopsy confirmation of the majority of the images was not available, we added images from three public datasets (Table 1), resulting in 3,083 labeled images that we used to train and test the classification algorithm. Due to the lack of standard in the labels of these datasets, two dermatologists identified the totality of the lesions in the datasets according to the nature of the lesion: malignant or benign neoplasm. There were 53, 17, 131 and 39 different classes in SD-198, Derm7-pt, Dermatology Atlas and Sao Paulo City/ HIAE Teledermatology Project Datasets, respectively.

Malignant tumors comprised 18% of all the images and 82% were benign tumors. We splitted this dataset into training (80%), validation (10%) and testing (10%). We used the DenseNet architecture [32] with ImageNet pre-initialization varying the optimizers to train the ensemble. Training was performed for 100 epochs with a batch size of 32 and early stopping regularization to avoid overfitting. All images were resized to 224 × 224 pixels, the default size of this network architecture. We used the Softmax function to learn the targets. To deal with the class imbalance, each class was weighted according to its frequency, so that the cases of rare conditions contributed more to the loss function in training, and we used in-place data augmentation. We also took into account the imbalance bias in the prediction layer.

### 3.2 Prediction visual explanation

To assist PCPs in understanding the AI output, we added a HeatMap to the AI report. This HeatMap is created utilizing Grad-Cam [33], which is a technique to verify which areas of the image had the most influence in the algorithm decision, making it possible for the PCP to understand which parts of the lesion the algorithm considered most important for the classification.

## 4. Results

### 4.1 Implementation of AI algorithms in the patient flow of a primary care unit

Our informal survey with PCPs showed that our typical subject is: male (57%), 31 years of age, single, with no children, Brazilian, 7 years since graduation, Family Doctor, working in São Paulo city, 40 hours/week, assisting a mean of 431 patients/month, who does not work anywhere else, has been working in primary care for more than 3 years, but with no intention to stay in primary care for the next 5 years; believes that electronic medical records and AI would facilitate their work, and his motivation to work in primary care is to have a closer doctor-patient relationship (S1 File).

Once in the primary care unit, the PCP logs into the application using a username and password, and it collects the patient’s information and photographs as described above. The CAD system is applied to the photographs taken. For each lesion analyzed, the AI generates a report with four different tabs. The first tab is the main one, presenting the image of the suspected lesion and its respective Heat Map (Visual Grading); the predicted output of the suspected lesion being melanoma or malignant (in percentage); the diagnosis based on that probability; and a suggestion about how the lesion should be managed. There is also an open space for the physician to leave comments, suggestions or criticism.

If no dermoscopy is available in the primary care center, the patient’s lesion would be evaluated only by the clinical AI algorithm, with two possible outcomes: suspected malignancy or not. In the first scenario, the recommendation would be to refer the patient to a dermatologist to perform the dermoscopy and decide whether to biopsy/remove it or not. In the second scenario, the recommendation would be to follow-up on the lesion and schedule a new screening test in one year, for example, or earlier, depending on the PCP’s criteria.

If the dermoscopy is available in the primary care unit, the PCP would follow the previous flow, but also include a dermoscopy image as the third photograph. The dermoscopy AI report would also show the same information as the clinical AI report, with the difference that instead of two possible outcomes, there would be three: high, moderate or low probability of being melanoma (Fig 2). Fig 3 shows a diagram indicating the suggested management for each type of model output. When the lesion is considered high probability, the suggested management given by the CAD system is to refer the patient to biopsy; for moderate probability, the protocol includes short term sequential dermoscopy in case of suspicious lesions (3 months and 6 months); and for low probability, a reevaluation in 12 months is advised. For low and moderate probabilities, an observation saying that if the lesion enlarges, changes color, bleeds or shows signs of inflammation, the patient should return for a new evaluation before the stipulated time. To ensure quality control of the photograph technique to be used by PCPs, we have made a tutorial video and created a tab in the app trying to orient the clinicians on how to take pictures. We are also studying the development of an algorithm to be used while the picture is being taken, that would interact with the individual, asking for new pictures if they do not have enough quality.

### 4.2 Algorithms performance

**Fig 4:**
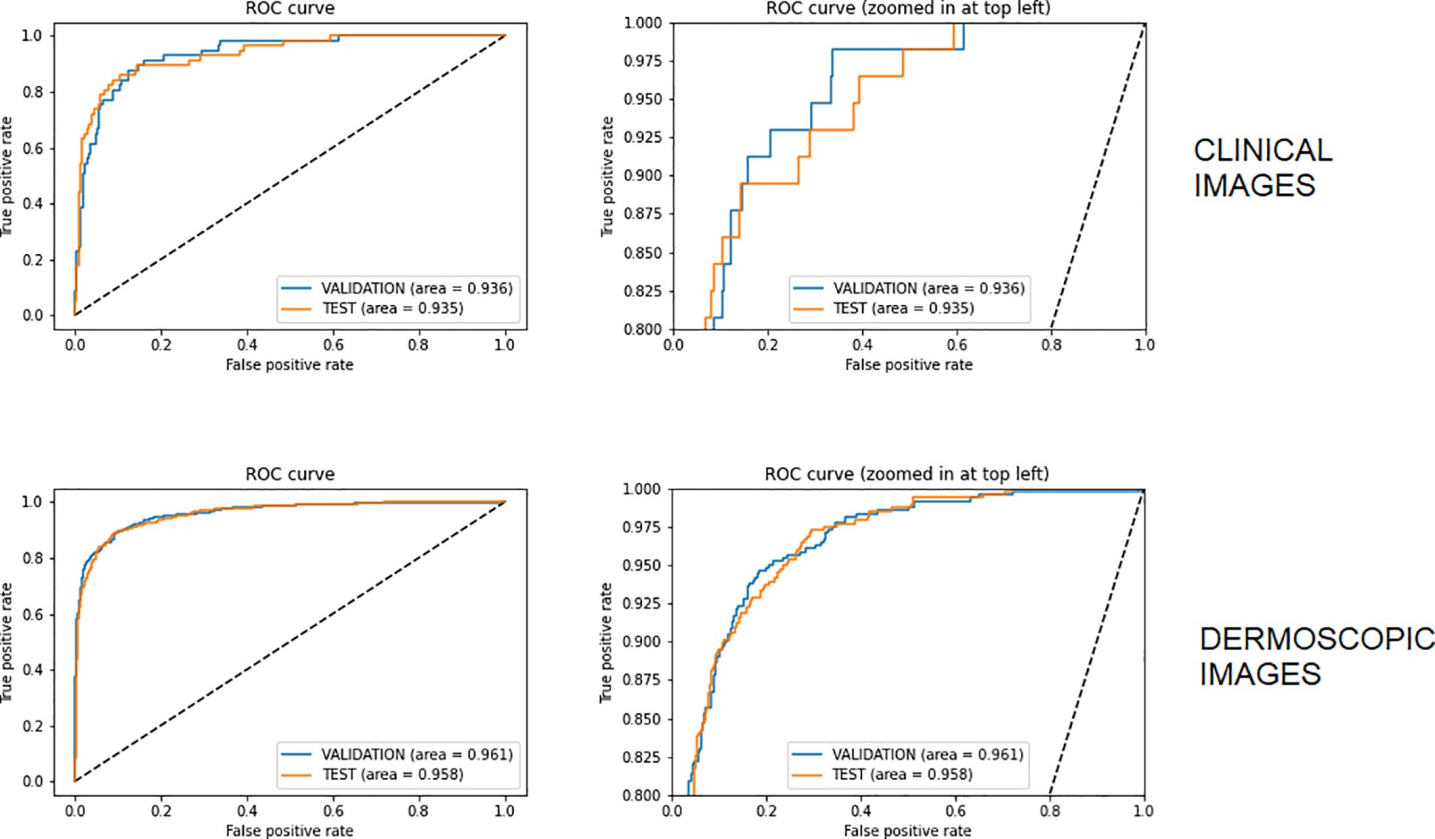
Receiver Operating Characteristic Curve (ROC) for clinical and dermoscopic images algorithms. Graphics on the right represent the same curves as the left, with zoomed in at the top left.

For the dermoscopy model, the segmentation algorithm achieved a mean average precision of 99.7%. In the clinical pipeline, the similarity filter model achieved an accuracy of 0.92 with 0.96 sensitivity and 0.98 specificity. Both clinical and dermoscopy models achieved accuracies higher than 0.8 and an AUC above 0.9. The number of incorrect classified melanomas (false negatives) were below 3% of all the images in the test dataset. Table 2 shows the performance of these algorithms measured by the most common metrics used in the literature. S1–S3 Tables show the results for models combined in the dermoscopy, clinical and similarity algorithms, respectively. Fig 4 shows Receiver Operating Characteristic Curve (ROC) curves for both models.

**Table 2:** Performance of the algorithms in validation and in test steps.

	VALIDATION		TEST	
Metric	Clinical Algorithm	Dermoscopic Algorithm	Clinical Algorithm	Dermoscopic Algorithm
Accuracy	0.8543	0.8895	0.8603	0.8928
Sensitivity	0.9122	0.9026	0.8947	0.9056
Specificity	0.8412	0.8866	0.8525	0.8900
True Positive	52/309	436/2635	51/308	432/2633
True Negative	212/309	1908/2635	214/308	1919/2633
False Negative	5/309	47/2635	6/308	45/2633
False Positive	40/309	244/2635	37/308	237/2633
Positive Predictive Value (PPV)	0.5652	0.6412	0.5795	0.6457
Negative Predictive Value (NPV)	0.9769	0.9760	0.9727	0.9771
AUC- ROC*	0.936	0.961	0.935	0.960

*AUC-ROC: Area Under the Receiver Operating Characteristic Curve

## 5. Discussion

### 5.1 Implementation of AI algorithms in the patient flow of a primary care center

There are three important questions to be asked when developing and implementing a CADS for skin cancer screening. First, for which type of image is it built for: clinical or dermoscopic? Second, is it intended to be a support tool for dermatologists or to be used for cancer screening by non-specialists, such as PCPs? Third, is it the goal to have it output the most probable diagnosis or to endorse a management?

We developed algorithms for both clinical and dermoscopic images to be used as a cancer screening tool for PCPs with the intention to guide lesion management. As far as we know, this is a new proposal in the literature and it is especially helpful when access to a dermatologist can be difficult or time consuming. The use of AI systems as a screening tool for the PCP’s assessment can enable risk stratification of lesions and/or individuals and dramatically improve timely access to specialist care for those requiring urgent attention.

Regarding the third point, one could argue that, if you have the most probable diagnosis, one would, consequently, also have the right management. In our opinion, since the gold standard diagnosis for skin cancer is still the histological report, it is more beneficial to focus on providing the correct lesion management plan than instead of the supposed corrected diagnosis. Most of the studies up to this point have focused on supporting diagnosis of dermatologists, and these studies have shown that dermatologists are the professionals that least benefited from using AI [33]. We expect to conduct a future clinical trial in which physicians will not be able to see the results of AI and do not interfere in dermatologists’ management. Although clinical pictures of certain regions were not included in the Dataset, we will not exclude any body site, as we are going to compare the results between AI and dermatologists or histopathological reports blindly, to assess its performance. Actually, we are already working on the app to include detailed parts of the face in the photograph protocol, in close-up, which will probably allow the similarity algorithm to be used for the face. If the performance was approved, then we expect to perform another clinical trial with the AI results displayed for the PCPs. Our CAD system will also make management suggestions for PCPs, but we are not planning to ensure consistency in this matter just yet. Legally, in Brazil, physicians are ultimately responsible for the patient management. Thus, we will have to gain trust and prove that our CAD system is a good, safe and reliable tool, before trying to ensure consistency in the management.

The description of the report generator in detail above, its content, and the lesion management suggestions for the PCP have not been discussed in previous studies. One study tested four ways of generating reports for AI, and concluded that, for a multiclass prediction, showing the predicted probability for each class was preferred among the other options [34]. The use of a Heat Map in the main page of our report generator is an interesting innovation (S2A and S2B Fig in S2 File), and we believe that assessing the clinical and/or dermoscopy image and its visual explanation makes the process more reliable for PCPs and patients, as does the explanation of the uncertainty around any algorithm. However, despite all the data generated by the AI algorithms, we truly believe that the physicians’ opinion is extremely important, since the final decision will be his/hers. In fact, for this reason, we aimed to develop our CADS to be used exclusively by physicians.

There are many other factors that play very important roles in the implementation of this new technology, besides having an accurate algorithm. There are barriers such as internet access, still nowadays, in many places around the world, including in Brazil that need to be addressed. Also, the adherence of the physicians to the photograph protocol and to the use of the app and the dermoscopy can be low. Therefore, even if we had the best algorithm, if it is not correctly used, it does not change anything in practice. Patients can be resistant to AI technology too. Thus, our next steps in this matter is to perform a small clinical trial in the real world, associated with ten PCPs, to gather much data information through the app and personal feedback about the usability and adherence to our algorithms.

### 5.2 Algorithms performance

Several studies have shown that CNNs trained on retrospective image data are capable of classifying skin cancer with sensitivities and specificities equal or superior to that of dermatologists [11–13, 34]. Clinicians with less experience gain most from AI support under experimental conditions [34]. Our algorithms have shown good metrics in general, and we believe that the use of two algorithms in sequence possibly increases the accuracy and, consequently, the trust in the process. The accuracy, around 84–89% for clinical and dermoscopic images respectively, was similar to other studies, ranging from 72–94%. Sensitivity was high for both systems, close to 90%, also compatible with the literature, which ranged from 81–95%. Specificity was 83% and 89% for clinical and dermoscopic images, showing good results as compared to the 70–93% in other studies. The area under the curve (AUC) obtained results of 92,6% and 96,0% for clinical and dermoscopic algorithms respectively, which was the best result for the dermoscopic images presented among many papers (85–96%).

### 5.3 Limitations

Physicians do need to be aware of the limitations of this CAD system and how to interpret its output. First, in the development of these algorithms, datasets were limited, which may affect their performance in real world implementation [35]. Specially for the clinical algorithm, many body parts were left out by our filter due to the difficulty encountered by the AI algorithm to recognize the target lesion in regions such as the face, hands, feet, and scalp or due to the images of the lesions not being collected properly following the collection protocol, which were classified as “non-ideal”. We expect to improve algorithm performance and include more body parts as new adequately-collected images that are incorporated to the dataset.

Although the use of both IA algorithms is promising, we do not have a “real world” study yet to draw any conclusions from. Nevertheless, there is a potential risk of over-reliance on AI. Physicians are more likely to change their minds if they are young, have little experience, are uncertain of a diagnosis and the algorithm provides a conflicting result [33]. It is important to consider how an algorithm might convey uncertainty to avoid false guidance, and this was one of the reasons why we included a page in the report to explain this possibility.

Another important aspect is regulation. Some countries, such as Australia, have developed an action plan to improve the processes by which new devices are approved for use [36]. In addition, there are international groups, such as the International Medical Device Regulators Forum, aiming to establish better processes for medical device regulation globally, including in Brazil. We do need to validate this new technology first in a blind way, and then, if the performance in “real world” is considered adequate, we need to study how it will influence the physicians’ decisions and how the outcome of the patient is affected. All technologies in the medical area ought to be rigorously tested before the implementation and intensely surveilled afterwards. The responsibility for patient care remains with the physician and, as such, a high level of continuous medical education must be performed. Nonetheless, artificial intelligence in dermatological lesions is likely destined to become a powerful tool in skin cancer assessment.

## Supporting information

S1 File PCP’s questionnaireu. (DOCX)

S2 File Details of AI report. (DOCX)

S1 Table Results for each of the five EfficientNetB6 models combined in the dermoscopy model. (DOCX)

S2 Table Results for each of the three DenseNets models combined in the clinic model. (DOCX)

S3 Table Results for each of the sixteen convolutional nets tested in the similarity filter algorithm. (DOCX)
