# Supplementary material for: Implementation of artificial intelligence algorithms for melanoma screening in a primary care setting
Source: PLoS One. 2021 Sep 22;16(9):e0257006. doi: 10.1371/journal.pone.0257006 (PMC8457457; doi:10.1371/journal.pone.0257006)
Supplement: S1 File — (DOCX) [file pone.0257006.s001.docx]

**S1 File. PCP’s Questionnaire**

Questionnaire used in 10 PCPs to build a profile and main results:

- sex: male (57%)
- age: 31 years old (mean)
- marital status: single (86%)
- number of children: 0 (100%)
- nationality: Brazilian (86%)
- years since graduation in Medicine: 7 years (mean)
- specialty: Family Medicine (57%)
- city and state where you work: São Paulo, SP (86%)
- hours of work/week: 40 (86%)
- number of patients/month: 431(mean)
- do you work somewhere else?  no ( 57%)
- which are you aim difficulties at work?
- which of these technologies would you choose to facilitate your job? electronic medical record (43%); unified platform to consult patient’s exams (14%); artificial intelligence (43%)
- do you intend to keep on working in primary care attention for the next 5 years? No (86%)
- how long have you been working in primary care? more than 3 years (57%); 1-3 years (14%) and ≤1 year (29%)
- what motivates you to work in primary care? To have a close doctor-patient relationship (72%)
