# Supplementary material for: Implementation of artificial intelligence algorithms for melanoma screening in a primary care setting
Source: PLoS One. 2021 Sep 22;16(9):e0257006. doi: 10.1371/journal.pone.0257006 (PMC8457457; doi:10.1371/journal.pone.0257006)
Supplement: S2 File — (DOCX) [file pone.0257006.s002.docx]

**S2 File. Details of AI report**

Second Tab: Understanding the Heat Map.

The "Heat Map" is a way of representing information through colors. Warm colors such as red, orange, and yellow are those that generally represent the points of greatest interest or intensity, and cold colors, such as green and blue, are the least important or least significant points. Through the color representation in the image analyzed by artificial intelligence (AI), we can show which areas the AI is "looking at" and which ones it considered most important for the final diagnosis (Fig. S2AB).

**S2A Fig. Examples of clinical images and their “Heat Maps”, with their respective suggested lesion management**. First row shows an image classified by the algorithm as suspicious of malignancy (left), with its respective “Heat Map” (right) and the management suggested. Second row presents a clinical lesion classified by the algorithm as non-suspicious of malignancy (left), with its respective “Heat Map” (right) and the management suggested.


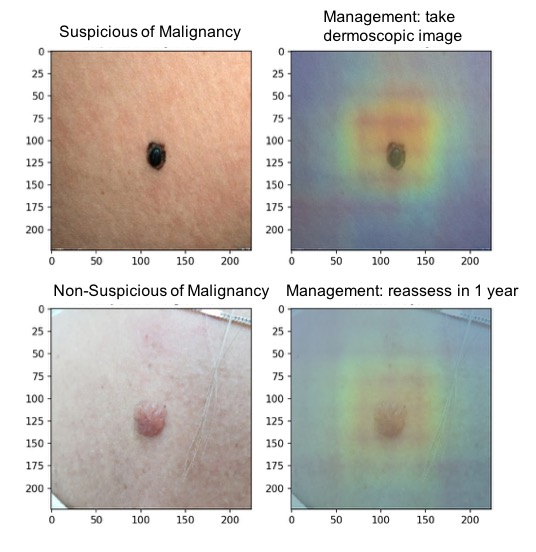


**S2B Fig. Examples of dermoscopic images and their “Heat Maps”, with their respective suggested lesion management.** Below on the left, we see the brownish lesion of interest in the upper left quadrant. In the image on the right, we observe that the AI detected the target lesion (in red) and considered the rest of the skin less important (in blue).


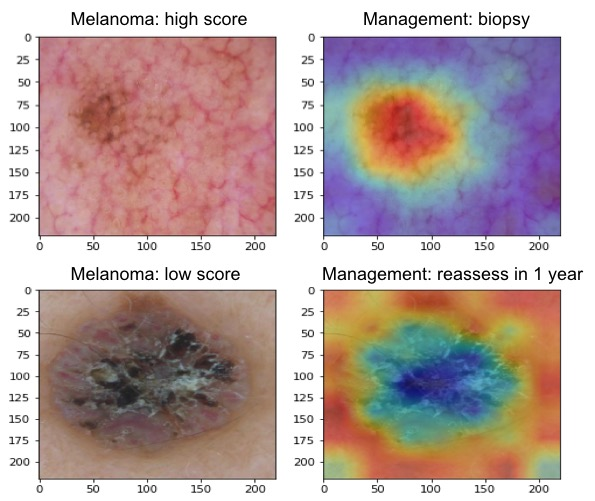


In the examples above, we illustrated how AI points where the lesion of interest is situated. Furthermore, when analyzing several dermoscopic images classified as "melanoma or" non-melanoma ", we observed a different "heat map" pattern for these classes. "Melanomas" appear with the lesion in warm tones (upper image), while "non-melanomas" appear with the lesion in cold tones and the bottom in red, as shown in the bottom image of the Fig. S2B.

Third tab: understanding the numbers in AI (probabilities and uncertainty)

The predicted output of the artificial intelligence (AI) algorithm has the purpose to show the physician what are the chances of that photographed lesion truly being a melanoma are. This value reflects the degree of confidence with which the artificial AI algorithm can diagnose melanoma. No algorithm reaches 100% of certainty. Therefore, there is always a degree of uncertainty in the diagnoses made by AI and this is fundamental in physicians' decision making.

AI uncertainty depends on the robustness of the algorithm. An AI algorithm is considered robust in relation to its predictions, if, regardless of how the image of the lesion is presented to the algorithm, the prediction, and consequently, the diagnosis, remains the same, it does not change. This means that, if the algorithm is not robust, an image classified as melanoma by the algorithm at one time, could be classified as non-melanoma at another; for example, if that same image were rotated 180 degrees. One way to minimize this type of uncertainty is to train the AI ​​algorithm with a large number of images of lesions, using them in various ways, rotating them, slightly displacing them, etc.

In conclusion, uncertainty metrics can be extremely useful in the decision-making process, as it is possible, for example, to establish a maximum acceptable limit of uncertainty for the algorithm. In addition, with information about uncertainty, we can define whether we should seek additional information before effective decision-making.

Fourth Tab: Who we are

This tab describes the project number, authors and affiliations.
