# Supplementary material for: Implementation of artificial intelligence algorithms for melanoma screening in a primary care setting
Source: PLoS One. 2021 Sep 22;16(9):e0257006. doi: 10.1371/journal.pone.0257006 (PMC8457457; doi:10.1371/journal.pone.0257006)
Supplement: S1 Table — (DOCX) [file pone.0257006.s003.docx]

**S1 Table. Results for each of the five EfficientNetB6 models combined in the dermoscopy model.**

| **Approach** | **Hyperparameters** | **accuracy** | **loss** | **sensitivity** | **specificity** | **TP** | **TN** | **FN** | **FP** |
| --- | --- | --- | --- | --- | --- | --- | --- | --- | --- |
| Data augmentation with GAN | class_weight & focal_loss | 0.87 | 0.02 | 0.91 | 0.86 | 435 | 1854 | 42 | 302 |
|  | class_weight & binary_crossentropy | 0.93 | 0.22 | 0.82 | 0.95 | 393 | 2045 | 84 | 111 |
| Original Data | class_weight & focal_loss | 0.82 | 0.03 | 0.93 | 0.80 | 445 | 1704 | 32 | 452 |
| Data Augmentation with conventional techniques | flip aug - class_weight & focal_loss | 0.84 | 0.03 | 0.93 | 0.83 | 444 | 1779 | 33 | 377 |
|  | mix aug - class_weight & focal_loss | 0.84 | 0.03 | 0.93 | 0.82 | 443 | 1773 | 34 | 383 |

TP: true positive; TN: true negative; FN: false negative; FP: false positive
