# Supplementary material for: Implementation of artificial intelligence algorithms for melanoma screening in a primary care setting
Source: PLoS One. 2021 Sep 22;16(9):e0257006. doi: 10.1371/journal.pone.0257006 (PMC8457457; doi:10.1371/journal.pone.0257006)
Supplement: S2 Table — (DOCX) [file pone.0257006.s004.docx]

**S2 Table. Results for each of the three DenseNets models combined in the clinic model.**

| **Approach** | **Hyperparameters** | **accuracy** | **loss** | **sensitivity** | **specificity** | **TP** | **TN** | **FN** | **FP** |
| --- | --- | --- | --- | --- | --- | --- | --- | --- | --- |
| DenseNet201 | class_weight & adam - without fine tuning | 0.66 | 0.87 | 0.91 | 0.60 | 52 | 152 | 5 | 99 |
| DenseNet169 | class_weight & rmsprop | 0.88 | 0.44 | 0.78 | 0.90 | 45 | 227 | 12 | 24 |
| DenseNet201 | class_weight & adam | 0.84 | 0.49 | 0.89 | 0.82 | 51 | 208 | 6 | 43 |

TP: true positive; TN: true negative; FN: false negative; FP: false positive
