# Supplementary material for: Implementation of artificial intelligence algorithms for melanoma screening in a primary care setting
Source: PLoS One. 2021 Sep 22;16(9):e0257006. doi: 10.1371/journal.pone.0257006 (PMC8457457; doi:10.1371/journal.pone.0257006)
Supplement: S3 Table — (DOCX) [file pone.0257006.s005.docx]

**S3 Table. Results for each of the sixteen Convolutional Nets tested in the similarity filter algorithm**

|  | Convolutional Net | Number of features | Final Loss | number of epochs | total accuracy | sensitivity | specificity | Model’s size MB  (Convolutional Net/ KNN) |
| --- | --- | --- | --- | --- | --- | --- | --- | --- |
| 1  2  3 | VGG16 | 1024  2048  4096 | 0,11  0,13  0,16 | 84  70  54 | 0,922  0,931  0,921 | 0,96  0,95  0,94 | 0,979  0,987  0,979 | 310  (154/156)  563  (252/311)  1071 (448/623) |
| 4  5  6 | VGG19 | 1024  2048  4096 | 0,14  0,15  0,14 | 105  99  106 | 0,90  0,904  0,901 | 0,92  0,92  0,90 | 0,977  0,975  0,982 | 331  (175/156)  583  (272/311)  1091 (468/623) |
| 7  8  9 | MobileNet | 1024  2048  4096 | 0,01  0,011  0,13 | 79  72  73 | 0,91  0,913  0,911 | 0,91  0,9  0,91 | 0,977  0,979  0,975 | 364  (208/156)  715  (404/311)  1419 (796/623) |
| 10  11  12 | MobileNetv2 | 1024  2048  4096 | 0,021  0,022  0,028 | 63  65  56 | 0,907  0,908  0,91 | 0,92  0,91  0,91 | 0,981  0,983  0,978 | 409  (253/156)  810  (499/311)  1612 (989/623) |
| 13  14  15 | NasNetMobile | 1024  2048  4096 | 0,039  0,068  0,046 | 126  89  123 | 0,881  0,90  0,891 | 0,89  0,91  0,90 | 0,972  0,974  0,977 | 375  (219/156)  732  (421/311)  1448 (825/623) |
